# Prognostic Signature of Chronic Kidney Disease in Advanced Age: Secondary Analysis from the InGAH Study with One-Year Follow-Up

**DOI:** 10.3390/biom12030423

**Published:** 2022-03-09

**Authors:** Anna Maria Meyer, Lena Pickert, Annika Heeß, Ingrid Becker, Christine Kurschat, Malte P. Bartram, Thomas Benzing, Maria Cristina Polidori

**Affiliations:** 1 Ageing Clinical Research, Department II of Internal Medicine and Center for Molecular Medicine Cologne, Faculty of Medicine and University Hospital Cologne, University of Cologne, 50937 Cologne, Germany; anna.meyer@uk-koeln.de (A.M.M.); lena.pickert@uk-koeln.de (L.P.); annika.heess@uk-koeln.de (A.H.); christine.kurschat@uk-koeln.de (C.K.); malte.bartram@uk-koeln.de (M.P.B.); thomas.benzing@uk-koeln.de (T.B.); 2 Institute of Medical Statistics and Computational Biology, Faculty of Medicine and University Hospital Cologne, University of Cologne, 50937 Cologne, Germany; ingrid.becker@uni-koeln.de; 3 Cologne Excellence Cluster on Cellular Stress-Responses in Aging-Associated Diseases (CECAD), Faculty of Medicine and University Hospital Cologne, University of Cologne, 50937 Cologne, Germany

**Keywords:** chronic kidney disease, frailty, kidney transplantation, laboratory signature, prognosis, renal replacement therapy (RRT)

## Abstract

The negative impact of chronic kidney disease (CKD) on health status and quality of life in older patients has been well documented. However, data on frailty trajectories and long-term outcomes of older CKD patients undergoing structured Comprehensive Geriatric Assessment (CGA) with multidimensional frailty evaluation are sparse. Here, we analysed records from 375 CKD patients admitted to our university hospital (mean age 77.5 (SD 6.1) years, 36% female) who had undergone a CGA-based calculation of the frailty score with the multidimensional prognostic index (MPI) as well as follow-up evaluations at 3, 6 and 12 months after discharge. Based on the MPI score at admission, 21% of the patients were frail and 56% were prefrail. MPI values were significantly associated with KDIGO CKD stages (*p* = 0.003) and rehospitalisation after 6 months (*p* = 0.027) and mortality at 3, 6 and 12 months (*p* = 0.001), independent of chronological age. Kidney transplant recipients (KTR) showed a significantly lower frailty compared to patients with renal replacement therapy (RRT, *p* = 0.028). The association between frailty and mortality after 12 months appeared particularly strong for KTR (mean MPI 0.43 KTR vs. 0.52 RRT, *p* < 0.001) and for patients with hypoalbuminemia (*p* < 0.001). Interestingly, RRT was per se not significantly associated with mortality during follow up. However, compared to patients on RRT those with KTR had a significantly lower grade of care (*p* = 0.031) and lower rehospitalisation rates at 12 months (*p* = 0.010). The present analysis shows that the large majority of older CKD inpatients are prefrail or frail and that the risk for CKD-related adverse outcomes on the long term can be accurately stratified by CGA-based instruments. Further studies are needed to explore the prognostic and frailty-related signature of laboratory biomarkers in CKD.

## 1. Introduction

**Figure 1:**
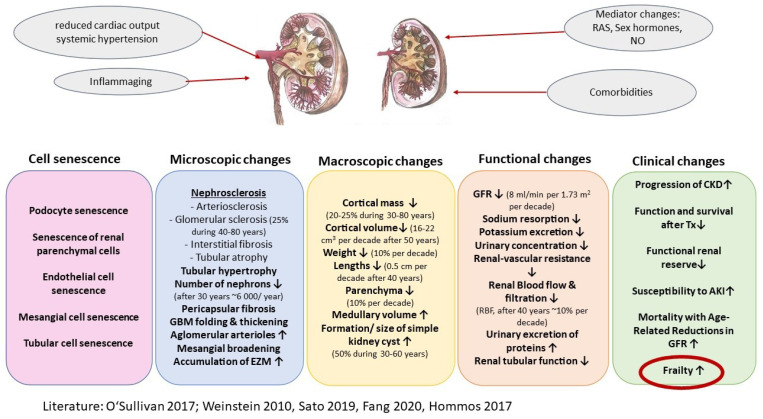
Structural and functional changes in the aging kidney. Cell senescence leading to microscopic and macroscopic changes imply changes in kidney function. These are accompanied by clinical changes. (Modified from [1,4,18,19,20]), EZM: extra cellular matrix; GFR: glomerular filtration rate; CKD: chronic kidney disease; AKI: acute kidney injury; RAS: renin-angiotensin-system; NO: nitrogen).

As life expectancy continues to improve, kidney aging has become an important challenge in clinical practice [1]. In an intra- and interindividual heterogenous way, kidney function decreases with increasing age, mainly due to vascular stiffening and fibrosis. Kidneys are among the organs with the most prominent changes during aging [2,3], some of the latter being associated to pathologic manifestations and others being part of the physiological aging process [4]. A decline in total nephron size and number, tubulointerstitial changes, glomerular basement membrane thickening and increased glomerulosclerosis (nephrosclerosis) are observed [4]. In addition, altered haemodynamic, physiologic and transcriptomic behaviour at rest impact on response to renal insults [2]. As a consequence, the ability of the kidney to withstand and recover from injury declines with age and the risk of developing progressive chronic kidney disease (CKD) increases [4]. CKD is a complex condition generally arising from a disordered kidney filtration barrier within glomeruli and is defined as damage of the glomerular filter (i.e., albuminuria) or decreased kidney function (i.e., glomerular filtration rate [GFR] <60 mL/min per 1.73 m^2^) lasting 3 months or longer, irrespective of clinical diagnosis [5,6] (Figure 1). CKD and its management are classified according to the 2012 KDIGO (Kidney Disease: Improving Global Outcomes) CKD guidelines; disease severity stages base upon GFR and albuminuria [7]. Due to the recent demographic change and predicted steep increase of the oldest-old population [8], CKD is a global public health priority. CKD affects more than 10% of the world’s population [5] and patients with CKD account for 20% of all medicare expenditures in people over 65 years of age [9,10]. In addition to the age-related changes mentioned above, the causes of CKD are as heterogeneous as aging itself and affect the kidney structure and function, such as hypertension, diabetes and hyperlipidaemia. In turn, CKD is associated with an increased risk of cardiovascular diseases, overall mortality, end-stage renal disease (ESRD) [11,12,13,14], frailty as well as high hospitalisation rates and disability [15,16,17].

In the past recent years, effective treatment options have been developed which can prevent the progress to renal failure in addition to reduce complication rates and the risk of cardiovascular disease and, therefore, improving survival and quality of life [21,22,23,24,25]. In advanced age, however, the success of interventions is often limited by overall frailty, disability and geriatric syndromes [26].

Therefore, personalised strategies tailored to patient’s functional status and risk are highly warranted [5,17,27,28]. Tailored interventions need to be established upon a solid base of evidence, but there is a substantial lack of data on the long-term outcomes of clinically well-characterised older CKD patients. To close this gap of knowledge, the present analysis aimed at investigating the overall frailty status and prognosis of older CKD inpatients treated in a highly specialised Nephrology unit and undergoing a structured Comprehensive Geriatric Assessment (CGA) with calculation of prognosis and multidimensional frailty according to a highly validated tool, the Multidimensional Prognostic Index (MPI) [29,30,31,32]. 

## 2. Material and Methods

### 2.1. Patients and Methods

**Figure 2:**
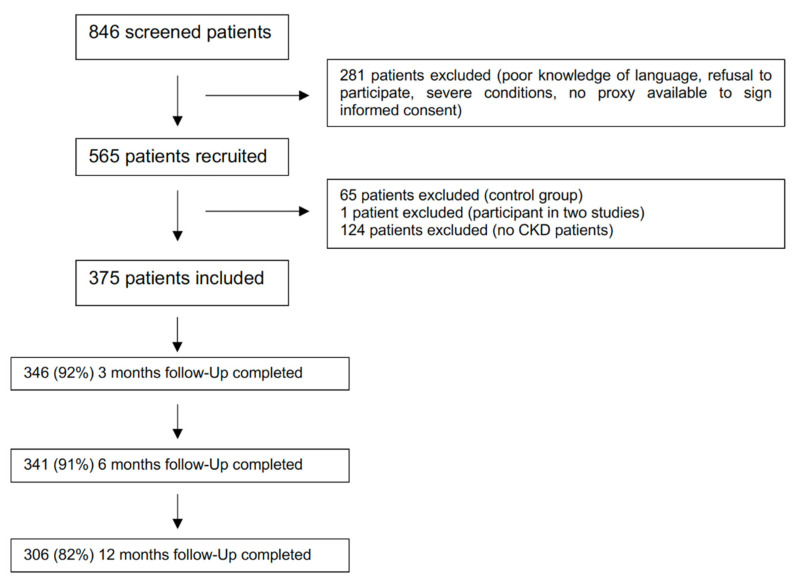
Flowchart.

The data presented here result from the secondary analysis of the MPI-InGAH study, which was conducted between June 2016 and July 2020, as previously described [28,33,34]. In this prospective, observational study, a total of 565 patients were recruited (Figure 2). This study was conducted according to the World Medical Association’s 2008 Declaration of Helsinki, the guidelines for Good Clinical Practice and the Strengthening the Reporting of Observational Studies in Epidemiology (STROBE) guidelines. The Ethical Committee of the University Hospital of Cologne approved the study (EK 16-213). All patients (or proxy respondents, when medical record indicated incapacity to give informed consent) signed informed consent to participate. Recruitment was carried out at the Department of Nephrology, Rheumatology, Diabetology and General Internal Medicine of the University Hospital of Cologne. Inclusion criteria were age over 65 years, multimorbidity (defined as coexistence of multiple (two or more) conditions, requiring long-term treatment [35]) and a hospitalisation period longer than four days. Exclusion criteria were refusal to participate in the study, language barrier and a hospitalisation period of less than four days. Patients underwent a CGA with a prognosis calculation using the MPI as described before [32]. Briefly, Activities of Daily Living (ADL) [36], Instrumental Activities of Daily Living (IADL) [37], Mini-Nutritional Assessment-Short Form (MNA-SF) [38], Short Portable Mental Status Questionnaire (SPMSQ) [39], Cumulative Illness Rating Scale (CIRS) [40] and Exton Smith Scale (ESS) [41]—as well as number of drugs taken by the patient and living conditions—were collected to calculate the MPI, which generates continuous values between 0 and 1. These can be used to subgroup patients into MPI-1 (robust, 0.00–0.33), MPI-2 (prefrail, 0.34–0.66) and MPI-3 (frail, 0.67–1.00) classes, to inform about low (MPI-1), medium (MPI-2) and high (MPI-3) risk, respectively, of mortality, rehospitalisation, admission to long-term care facilities and increase of nursing needs within 1, 6 and 12 months after initial evaluation [42]. Additional information was collected regarding presence of 16 geriatric syndromes (GS) and 11 resources (GR) [28], as well as their reciprocal relationships. Information on grade of care (GC, level of care and nursing needs according to the German nursing care insurance (grade 0 to 5, with 0 indicating no dependence [43])) was also available for all patients. All patients received follow-up calls 3, 6 and 12 months after discharge and were asked for the following information: mortality, length of hospital stay (LHS), GC, institutionalisation, number of medications, rehospitalisation and home care.

Furthermore, data obtained in the context of curative care such as laboratory data (blood and urine samples) were evaluated retrospectively for blood on admission (±3 days) and on discharge (±3 days); for urine, we only collected one sample—if several samples were preserved, we only analysed the first sample.

### 2.2. Data for Present Secondary Analysis

In the present secondary analysis, patients were included if they (1) had a diagnosis of CKD, defined as kidney damage or eGFR < 90 mL/min/1.73 m^2^ for 3 months or more, irrespective of cause [5], or were kidney transplant recipients (KTR), and (2) had undergone CGA during hospitalisation. Of the 565 screened patients, 375 met the criteria and were included for further analysis (Figure 2). The clinical information on kidney disease status was based on hospital records, with the main requirement of availability of CKD stage according to the KDIGO classification (KDIGO G2: GFR 60–89 mL/min/1.73 m^2^, G3a: GFR 45–59 mL/min/1.73 m^2^, G3b: GFR 30–44 mL/min/1.73 m^2^, G4: GFR 15–29 mL/min/1.73 m^2^, G5: GFR < 15 mL/min/1.73 m^2^; A1: albuminuria < 30 mg/g creatinine, A2: albuminuria 30–300 mg/g creatinine, A3: albuminuria > 300 mg/g creatinine [44]). On the basis of the latter, patients requiring renal replacement therapy (RRT) were recorded as belonging to stage G5. CKD cause, laboratory values on admission and discharge as well as comorbidities and concomitant medications were collected. If patients had required RRT, pre- or in-hospital baseline was recorded, including information on access—venous or peritoneal catheter vs. vascular access (shunt)—as well as on type of RRT (haemodialysis (HD) vs. peritoneal dialysis (PD)). If patients were KTR, the type of donation (deceased donor, living donor) and the date of renal transplantation were recorded. 

### 2.3. Statistical Analysis

Descriptive statistics are expressed using absolute numbers and relative frequencies for categorical variables and means (standard deviation, SD) or medians (quartiles (Q) Q1–Q3) for continuous variables.

Normal distribution was tested by Kolmogorov–Smirnov tests. Depending on distribution, continuous variables were compared by t-tests or non-parametric Mann–Whitney U tests between two groups, by Kruskal–Wallis tests between more than two groups. Rates were compared by Chi-square test or Fisher’s exact test. The variable “more GR than GS” was calculated by comparing the relative number of 16 GS with the relative number of 11 GR. If there were more GR than GS, this variable was rated “yes”. 

In Table 1, all CKD patients (*n* = 375) were subdivided according to their MPI risk group at admission (MPI-1 to MPI-3), *p*-values were calculated to test the association between MPI score and the tested variable and adjusted for age, gender and KDIGO G-stage with linear/logistic regression analysis. 

In Table 2, all CKD patients (*n* = 375) were compared based on KDIGO G-stage (G2-G5). Similarly, *p*-values were adjusted for age, gender and MPI with linear/logistic regression analysis.

Table 3 tested the association of patients undergoing RRT (*n* = 138) with HD or PD, which has been adjusted for MPI with linear/logistic regression analysis. 

To measure outcomes for KTR patients compared to patients undergoing HD-RRT (*n* = 175), Table 4 was adjusted by age with linear/logistic regression analysis. Table 5 compares patients with KDIGO stage G4-5 without HD and patients with HD (*n* = 190) and was adjusted for age with linear/logistic regression analysis. No adjustments were made if not otherwise specified. Survival length in MPI groups was calculated as time from recruitment to death or until last date observed. Survival time was analysed using the Kaplan–Meier estimator, and groups were compared by a log-rank test. Because KTR were significantly younger than patients on RRT, comparisons concerning KTR and RRT were adjusted for age. For one-year all-cause mortality, a receiver operator characteristic (ROC) curve was performed and the area under the curve (AUC) was calculated. Two-tailed probabilities were reported and a significant level alpha of 5% was used for each analysis.

All analyses were performed using SPSS (Statistical Package for Social Sciences, SPSS Inc., Chicago, IL, USA, version 27.0) software.

## 3. Results

### 3.1. CKD, Frailty and Long-Term Prognosis According to KDIGO

The clinical and demographic characteristics of the study population are shown in Table 1, as well as according to KDIGO in Table 2. Mean age was 77.5 (SD 6.1) years, and 133 patients (36%) were female. Based on the MPI-score at admission, 21% of the patients were frail and 56% prefrail.

**Figure 3:**
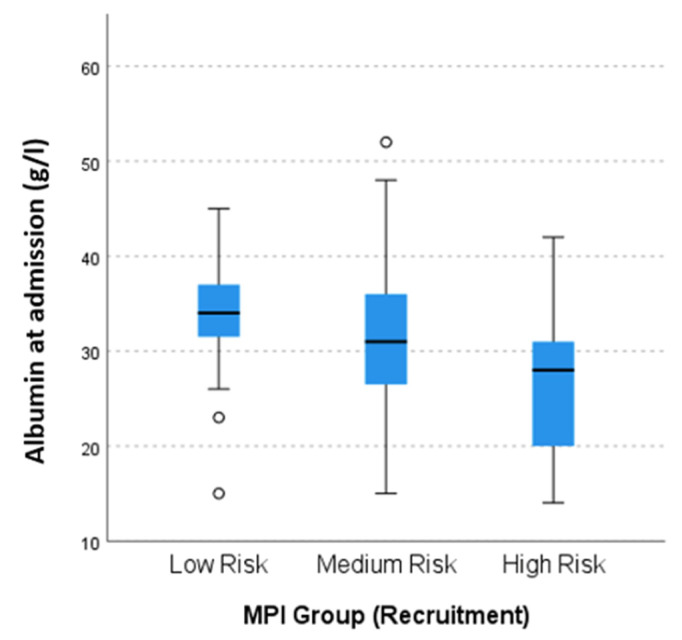
Albumin at admission according to MPI group. ° marks statistical outliers.

In the whole patient sample, higher age (*p* < 0.001), lower education length (*p* = 0.006), higher LHS (*p* = 0.002) and more falls (*p* = 0.009) in the previous 12 months, higher GC (*p* < 0.001) and use of home services (*p* < 0.001), higher number of Geriatric Syndromes (GS) (*p* < 0.001) and lower number of Geriatric Resources (GR) (*p* < 0.001) were significantly associated with a higher MPI score, indicating a higher frailty grade and poorer overall prognosis (Table 1). The BMI was significantly lower in in patients with a higher KDIGO stage (*p* = 0.012, Table 2). The analysis of laboratory parameters showed a strong association with frailty as assessed by the MPI value with lower total protein serum levels (*p* = 0.007) on admission, lower serum albumin levels on admission and discharge (*p* < 0.001, Figure 3), as well as with higher serum C-reactive protein (CRP) levels at admission (*p* = 0.016) and discharge (*p* < 0.020). After adjusting for age, gender, KDIGO-G stage and MNA-SF, serum albumin was still significantly associated with the MPI-score (*p* = 0.006). Additionally, patients with a higher KDIGO G-stage showed significantly lower Albumin (*p* = 0.049, Table 2).

**Figure 4:**
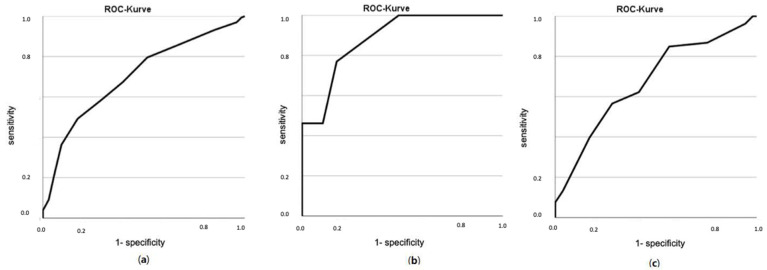
AUC area for one-year all-cause mortality. (**a**) All CKD patients with 0.71 (95% CI, 0.64–0.76); (**b**) KTR patients with 0.88 (95% CI, 0.78–0.98); (**c**) RRT patients with 0.67 (95% CI, 0.57–0.78).

**Figure 5:**
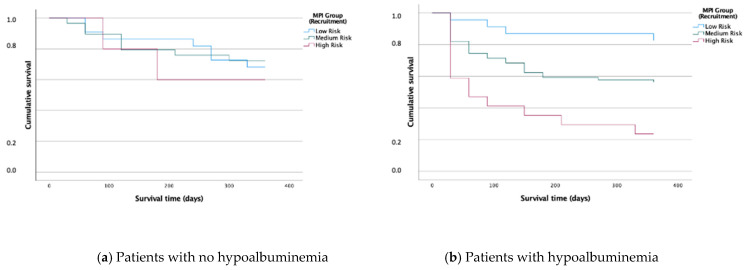
Cumulative Kaplan–Meier survival after 12 months in CKD patients according to MPI, compared to patients without hypoalbuminemia ((**a**), on the left) and with hypoalbuminaemia ((**b**), on the right).

In total, 75% of MPI-1 group patients, 61% of MPI-2 and 25% of MPI-3 (*p* = 0.001) were alive at 12 months FU, with an ROC area for one-year all-cause mortality of 0.71 (95% CI, 0.64–0.76, Figure 4a). Especially in the Kaplan–Meier curve for CKD patients with hypoalbuminaemia (albumin < 35 g/dL), the one-year survival was significantly different according to MPI group, with a higher MPI showing significantly higher mortality (*p* < 0.001, survival for hypoalbuminaemia patients with MPI-1: 82%, MPI-2 50%, MPI-3: 24%; Figure 5b). Hypoalbuminaemia was independently associated with the MPI (*p* = 0.002) but not with KDIGO G-stage (*p* = 0.086) adjusted for age, gender and each other. 

MPI values were significantly associated with KDIGO G-stages, with a higher MPI being associated with a higher KDIGO G-stage (*p* = 0.003, Table 2). 

Patients belonging to KDIGO stage G5 showed a significantly lower number of GR (*p* = 0.006) compared to patients with lower KDIGO G-stages. Polypharmacy was significantly more prevalent in higher KDIGO G-stages (*p* < 0.001). 

Rehospitalisation rates 6 (*p* = 0.038) and 12 months (*p* < 0.001) after initial evaluation were significantly associated with KDIGO G-stage, as well as GC after 6 (*p* < 0.001) and 12 months (*p* = 0.003). Mortality rates after 12 months were significantly higher in higher KDIGO G-stages (*p* < 0.001).

### 3.2. RRT Group: HD vs. PD

A total of 138 patients (37%, Table 3) received RRT, of which 11 patients (8%) underwent PD. Most patients (82%) undergoing PD lived together with relatives. Receiving RRT (*p* = 0.181, Table 1) or which kind of RRT (*p* = 0.457, Table 3) was not significantly associated with MPI score. A higher CIRS-Score was significantly associated with HD compared to PD (5.6 vs. 4.5, *p* = 0.026 adjusted for MPI). PD patients had significantly more emotional resources (*p* = 0.045) and less sensorial impairment (*p* = 0.019). At discharge, the MPI of PD patients was significantly lower, even when adjusted for MPI at admission and compared to HD patients (0.41 vs. 0.54, *p* = 0.021).

After one year, 50% of the RRT patient group were still alive, showing no significant difference in mortality in different RRT groups (*p* = 0.691). In total, 95% of RRT patients were rehospitalised during the FU period. More HD patients had a GC after 12 months compared to PD patients (73% vs. 25%, *p* = 0.083), although this effect was not statistically significant. The ROC area for one-year all-cause mortality according to MPI was 0.67 for patients undergoing RRT (95% CI, 0.57–0.78, *p* = 0.002, Figure 4c). 

### 3.3. KTR vs. RRT

Forty-four patients (12%, Table 4) of the sample were KTR. The mean time since kidney transplantation was 7.7 years (SD 8.0), with 51% deceased donor and 35% living donor (14% missing information).

Compared to patients undergoing HD-RRT, KTR patients were significantly younger (*p* < 0.001). KTR patients had experienced significantly fewer falls (*p* = 0.014) and had significantly lower GC (*p* = 0.031) than RRT patients. The MPI value was significantly lower in KTR compared to RRT patients (0.52 vs. 0.43, *p* = 0.028, Table 4). 

**Figure 6:**
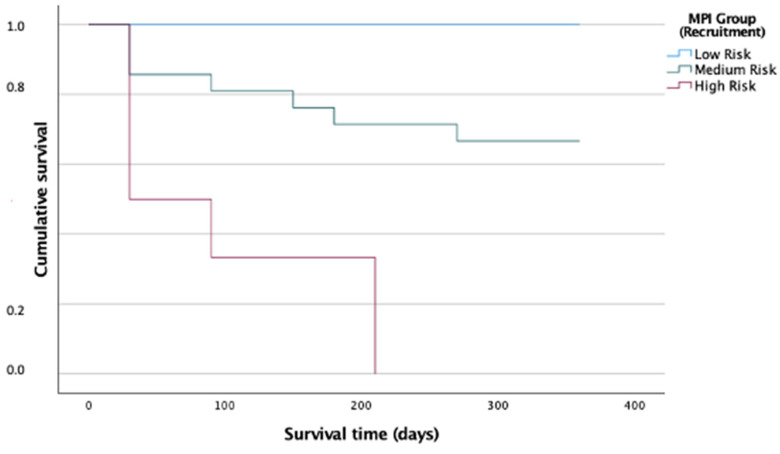
Cumulative Kaplan–Meier survival after 12 months in KTR patients according to MPI.

After 12 months of follow-up, 71% of KTR were still alive, compared to 49% of RRT patients, although this effect did not remain significantly different after adjusting for age and MPI (*p* = 0.395). KTR showed significantly less GC (*p* = 0.015) and a significantly lower rehospitalisation rate (*p* = 0.019) after 12 months compared to RRT patients. The Kaplan–Meier analysis for the cumulative survival time showed a significant difference between the MPI groups; all KTR in group MPI-1 survived, 50% of those survived in the MPI-2 group and none of those belonging to the MPI-3 group survived (*p* < 0.001, adjusted for age, Figure 6). The AUC for one-year all-cause mortality was 0.88 (95% CI, 0.78–0.98, Figure 4b). 

### 3.4. CKD KDIGO G4-5 Patients: No-RRT vs. HD-RRT

**Figure 7:**
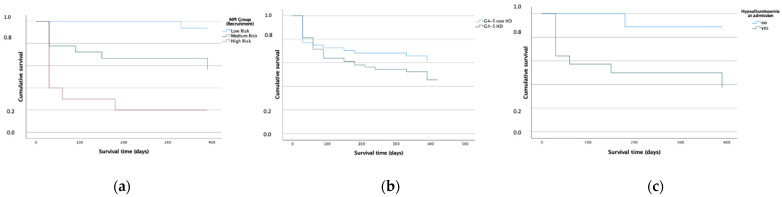
Cumulative Kaplan–Meier survival after 12 months in patients with KDIGO stage G4–5 without receiving renal replacement therapy (RRT) according to their MPI group at admission (**a**, on the left), according to whether they receive RRT or not (**b**, in the middle) and patients with KDIGO stage G4-5 without RRT according to hypoalbuminaemia ((**c**), on the right). (**a**) Patients with G4/5 and no dialysis according to MPI. (**b**) Patients with G4/5 according to chronic dialysis. (**c**) Patients with G4/5 and no dialysis according to hypoalbuminaemia.

Of the 201 patients with KDIGO G4-5, 52 (26%, Table 5) had not received any form of RRT (no-RRT). No-RRT patients were significantly younger than RRT recipients (*p* = 0.027). Compared to patients receiving HD-RRT, the LHS of no-RRT patients was significantly lower (12.0 vs. 20.4 days, *p* = 0.003). In addition, no-RRT patients showed a tendency to better MPI values compared to patients with HD-RRT (MPI 0.47 vs. 0.53, *p* = 0.052), as well as significantly less GS (*p* = 0.044) and a significantly higher BMI (*p* = 0.046). Regarding survival after 3 and 6 and 12 months, there was no significant difference between groups (*p* = 0.137, Figure 7b, no-RRT: 62%, HD-RRT: 45%). However, no-RRT patients showed a significantly lower prevalence of GC after 12 months (*p* = 0.003) compared to HD-RRT patients. Again, mortality rates were significantly associated with MPI values in no-RRT patients (*p* < 0.001, survival after 12 months: MPI-1: 94%, MPI-2: 56%, MPI-3: 20%, Figure 7a). Mortality risk was also associated with hypoalbuminaemia (*p* = 0.028, survival after 12 months: hypoalbuminaemia: 37%, no hypoalbuminaemia: 89%, Figure 7c).

## 4. Discussion

This secondary analysis from a relatively large-sized prospective evaluation of older CKD patients delivered several relevant observations which should be highlighted.

First, the multidimensional prognostic and frailty signature of CKD patients is strongly associated with the KDIGO-based classification of the renal impairment independent of age and gender. The MPI as a comprehensive prognostic tool [32] has been previously shown to improve the predictive value of GFR for outcomes of CKD patients [15,45]. If taking into consideration the MPI in its newly established role of comprehensive frailty index [30,31], its association with KDIGO can be discussed in the context of existing data on the association between higher frailty levels and higher KDIGO stage [44]. However, so far, previous studies linking CKD and frailty have only taken physical frailty into account [27,46], whereas the MPI is a feasible tool considering several factors, beyond organ illness, massively influencing prognosis—functions, mobility, cognition, nutrition, social aspects, multimorbidity and polypharmacy. The MPI shows that only a multidimensional consideration of the frailty makes it possible to map the prognosis. All eight domains of the MPI are, therefore, rated equally in the index. Of note, accordingly with the known high prevalence of physical frailty in CKD, in our sample, multidimensional—not only physical —prefrailty and frailty affect 77% of CKD patients, which is the large majority, also reflecting the strong, well established multifactoriality of both conditions [47]. Of note, it is being increasingly shown that multidimensional frailty indices are able to more accurately capture outcome risks of older patients compared to monodimensional phenotypes [48,49]. As the KDIGO guidelines recommend to assess routinely the prognosis of CKD patients using risk prediction instruments [50], currently, there are no uniform assessment standards or prognosis instruments for older patients, and thus, the MPI might represent a feasible instrument for this purpose. 

In this context, it is worth to mention that a lower education length (*p* = 0.006, Table 1) was significantly associated with a higher MPI score. Frailty risk has been shown to be associated to lower social class in childhood [51] and further studies might be directed at exploring specific factors of social inequalities associated to frailty in CKD to improve multidimensional early interventions.

Second, the present analysis reveals for the first time a profile of higher MPI-CKD patients. These more often chronologically older patients are frequently male, although the percentage of female patients increases across MPI groups, in agreement with data on the general frail population [52]. Of note in this context, while CKD is in general more prevalent in female than male patients, in our population, men are more represented, likely due to the known fact that men are more often affected by severe CKD stages, receive RRT more often than women and are, therefore, more often hospitalised [53]. In addition to being more often an older male person, the high MPI-CKD profile includes having fewer education years (*p* = 0.006), higher LHS (*p* = 0.002), more falls in the previous year (*p* = 0.009), higher nursing needs and home services (*p* < 0.001) and lower circulating levels of total proteins (*p* = 0.007) and albumin (*p* < 0.001) at admission than the low MPI-CKD profile (Table 1). The more frequent presence of heart disease, dementia, depression and peripheral artery disease in MPI-2 and -3 group patients than MPI-1 group patients was shown to be independent from age, gender and KDIGO, suggesting a predominant prognostic feature of these comorbidities beyond CKD severity.

To complete this picture, the high MPI-CKD profile is at high risk of mortality. Compared to Pilotto et al. [45], the one-year mortality rate in our sample was roughly twice as high for each MPI class (MPI-1 25% vs. 12%, MPI-2 39% vs. 21%, MPI-3 75% vs. 38%), which may be due to the different setting of a university hospital Nephrology unit with acutely very ill patients in need of high performance medicine compared to a geriatric unit. Overall, however, the heterogeneity of aging with a comparable ROC area for MPI and one-year all-cause mortality of 0.71 (95% CI, 0.64–0.76) vs. 0.70 (95% CI, 0.66–0.73) [45] could also be confirmed in our analysis (Figure 4a).

A similar view of the results obtained in the present analysis discloses the profile of high KDIGO stage carrier: higher rehospitalisation rates, (*p* < 0.001), higher nursing needs (*p* = 0.003) and mortality risk (*p* < 0.001) up to 12 months after hospital discharge with respect to lower KDIGO stage patients. The independence of these results from MPI is indicative of the strong impact of the CKD on the patients’ trajectories beyond their overall health status. Indeed, the significantly increased risk of physical frailty and mortality in older CKD patients [7,11,14,17,27,46,47,54,55]. However, the current KDIGO guidelines from 2012 do not take age into account when classifying the severity of CKD, although current research shows that from an age of about 45 years, the eGFR physiologically decreases by ~0.88 mL/min/1.73 m^2^/year [56]. This is particularly important because at the moment kidney aging is not relevantly differentiated from kidney disease, just as the severity of CKD in old age may be overestimated. Therefore, more and more scientists are calling for the CKD definition and also the KDIGO guidelines to include age-specific thresholds for GFR [57]. This could help preventing overdiagnosis and, thus, overtreatment for older people—but for younger patients, this could also enable earlier diagnosis at a time when preventing CKD is still possible.

A third main finding from the present analysis is that hypoalbuminemia and a high MPI score were independently significantly associated (*p* < 0.001). Previous reports of a “metabolic signature” of MPI in older, multimorbid patients [58,59] appears to be present in patients with CKD and especially ESRD. In our sample, especially for CKD patients with hypoalbuminaemia, the MPI showed a high prediction for survival time (*p* < 0.001). As shown in Table 1, the MPI score was significantly associated with serum albumin levels (*p* < 0.001) independent of age, gender and KDIGO stage and this significance increased in the further model adjusted also for MNA (*p* = 0.006). Due to the cross-sectional nature of the observed association between frailty and hypoalbuminemia, it is not possible by means of the present analysis to disclose the causal or epiphenomenal role of poor albumin-levels in frailty with or without CKD. Hypoalbuminaemia represents a signal of malnutrition [60] and is directly associated with the likelihood of developing frailty conditions [61]. Protein energy wasting (PEW) is known to be a common problem in patients with CKD and is known to be associated with adverse clinical outcomes, especially in individuals receiving maintenance RRT [62]. However, since hypoalbuminemia is also associated with sarcopenia, the latter may hinder food intake through reduced mobility, with the consequence of a poor, protein-deficient, diet. The higher rate of depression in CKD patients shown in our analysis (Table 1) and the associated loss of appetite could also be a modulating factor [63]. This could lead to a vicious cycle of lower albumin, poor nutrition and higher frailty in older CKD patients, who are already at risk of deranged homeostasis with negative body composition alterations, and they can act synergistically to cause an increased risk of mortality [64]. As aging and CKD are associated with systemic inflammation [65,66], it would also be interesting to investigate the association between inflammation markers (for example hs-CRP, IL-6, TNF, lipid peroxides and anti-oxidants) and the progression of frailty and CKD in a further prospective study. Although it has not been unequitable proved in studies yet, it is possible that nutritional interventions slow disease progression independently [67]. As there are no large randomised clinical trials that have tested the effectiveness of nutritional interventions on mortality and morbidity of CKD patients, further studies seem necessary to show the relationship between prognosis and nutrition, in particular to what extent the prognosis can be influenced by possible interventions to improve the prognosis, such as low-protein diet [68], chronic administration of nutritional supplementation [62] or amino-acid mixtures (particularly those enriched in branched-chain amino acids) [69]. 

In our analysis, patients in a late CKD stage (KDIGO G4-5) without dialysis (conservative therapy, no-RRT) had a comparable 12-month survival, a significantly lower rehospitalisation rate, a higher albumin level and a lower one MPI frailty compared to a patient on HD-RRT. These results might point out that at least two collectives of patients are combined in KDIGO stage 5: patients who have an urgent medical need to initiate dialysis have no other choice than to initiate RRT to prevent further, potentially lethal harm. On the other hand, patients who have a highly reduced, but rather stable kidney function might benefit from a conservative treatment, including regular nephrological controls, to initiate RRT based on sole calculations of the GFR, since RRT itself is associated with various complications. However even in KDIGO G5 there is a difference in frailty between with and without dialysis—this is one of the important results here. Thus, the MPI could both be used as an aid in clinical decision making as to whether dialysis therapy should be started (of course together with clinical parameters), and it could possibly also help for the decision of whether PD or HD is chosen. 

KTR patients had a significantly lower MPI than RRT patients (*p* = 0.028). Whether this prognostic significance of the MPI also applies to older KTR patients has not yet been shown. More and more studies show that kidney transplantation, even in older age (>65 years), has a strong beneficial influence on survival and quality of life of patients, especially compared to RRT patients, who have a strongly increased risk of frailty and sarcopenia and an increased mortality risk [70,71,72,73]. According to the literature, frailty status changes after kidney transplantation; it initially worsens directly after transplantation and then ameliorates—transplanted patients were most likely to show improvements in their physiological reserve, suggesting that pretransplant frailty is not an irreversible state of low physiological reserve [74]. In the present 12-month follow-up period, a significant association was found in the survival of transplanted patients and their MPI groups (*p* < 0.001, Figure 6); indeed, the MPI was strongly associated with one-year all-cause mortality with an ROC area of 0.88 (95% CI, 0.78–0.98). There is no current standard for selecting older patients for a transplant that contains a CGA, but it can be assumed that the examiner unconsciously uses the criteria of the CGA to decide, so that a selection bias (MPI-1 patients may be selected for a KTR more often) cannot be ruled out. With a MPI assessment, an additional criterion might be established that helps in the difficult decision of whether to add an older patient on the deceased donor waiting list, as well as whether to adds an early warning system in the follow-up of transplanted patients. Thus far, few examples are given in the literature where frailty instruments or a CGA are used to assess outcome or immediate post-operative complications before transplantation [75]. To assess individual benefits of a renal transplantation, a CGA with prognosis calculation like the MPI seems to be a suitable tool [76] and should even become part of the clinical routine for patients on the deceased donor waiting list or for patients who want to be admitted. 

There are several limitations of this study. First, this was a secondary, retrospective analysis of a prospective study cohort which was not recruited for this question; however, we could benefit from a very well-characterised cohort, especially with very accurately raised nephrological parameters. A second limitation is that this study was a cross-sectional study, although we had a one-year follow-up period; especially the nephrological parameters were only collected at one specific point of time.

The association between frailty status and multiple adverse outcomes suggests that exercise-based interventions to improve physical function and mobility may have far-reaching benefits in older adults with kidney disease [77]. Further studies, especially translational trials, are needed to characterise the relationship between kidney disease and frailty even better, and they are most important to identify opportunities to intervene. Physicians should fully disclose the risks of CKD and opportunities for treatment to patients of all ages and evaluate and manage cases according to the level of risk, even if this is challenging. Addressing healthy aging might make renal aging-associated frailty more preventable than inevitable.

## 5. Conclusions

This secondary analysis shows a multidimensional prognostic of signature for MPI frailty in CKD patients, which is strongly associated with the KDIGO G-stages. These findings indicate that an MPI assessment should be used in older CKD patients to determine the prognosis. Displaying the overall importance of nutrition in the frailty cascade of CKD patients, in this study malnutrition, and in particular hypoalbuminemia, were indicative of a poor prognosis and were associated with higher frailty—nutritional interventions, therefore, seem to be of enormous importance in CKD patients.

Furthermore, the initiation of RRT in CKD G4-5 and the kind of RRT showed in this study significant profiles according to MPI frailty and prognosis. Patients with CKD G4-5 might have, in some parts, better outcomes with conventional therapy, so that the decision to initiate RRT should be carefully considered. In addition, we showed that KTR patients had a significantly lower frailty compared to patients receiving RRT. These findings could indicate that an MPI assessment for RRT or KTR therapy decision making in older ESDR patients could be of outstanding importance, especially in order to avoid misjudgements due to advanced age.

## Figures and Tables

**Table 1:** Clinical and demographical characteristics of all patients according to their MPI group.

		Total *n* = 375	MPI-1 *n* = 86 (23%)	MPI-2 *n* = 210 (56%)	MPI-3 *n* = 79 (21%)	*p*-Value °
Demographic						
Age (years), mean (SD)		77.5 (6.1)	75.4 (5.5)	77.7 (5.7)	79.6 (7.0)	<0.001
Female, *n* (%)		133 (36)	21 (24)	81 (39)	31 (39)	0.091
LHS, median (Q1-3)		11 (6–19)	8 (5–15)	10 (6–18)	17 (10–26)	0.002
Education (years), median (Q1-3)		11 (10–14)	12 (11–15)	11 (10–14)	11 (9–12)	0.006
Admission status, *n* (%)	New admission to hospital	174 (47)	58 (69)	67 (32)	42 (53)	<0.001
	Transferred from internal ward	122 (33)	13 (16)	67 (32)	42 (53)	
	Transferred from external ward	74 (20)	13 (16)	44 (21)	17 (22)	
Hospitalisation last 12 months, *n* (%)		267 (71)	55 (64)	152 (72)	60 (76)	0.153
Falls last 12 months, *n* (%)		155 (41)	21 (24)	96 (46)	38 (48)	0.009
Comorbidities, *n* (%)						
Hypertension		263 (70)	57 (66)	145 (69)	61 (77)	0.188
Heart disease		270 (72)	48 (56)	165 (79)	57 (72)	<0.001
Cardiac arrhythmia		229 (61)	41 (48)	131 (62)	57 (72)	0.036
Diabetes mellitus		182 (49)	37 (43)	108 (51)	37 (47)	0.269
Chronic obstructive pulmonal disease		61 (16)	10 (12)	36 (17)	15 (19)	0.341
Dementia		14 (4)	1 (1)	5 (2)	8 (10)	0.033
Depression		29 (8)	5 (6)	12 (6)	12 (15)	0.048
Peripheral artery disease		81 (22)	10 (12)	51 (24)	20 (25)	0.012
Eye disease		65 (17)	7 (8)	42 (20)	16 (20)	0.031
Geriatric profile						
MPI-value admission, mean (SD)		0.48 (0.17)	0.27 (0.05)	0.49 (0.09)	0.74 (0.06)	-
MPI-value discharge, mean (SD)		0.47 (0.15)	0.30 (0.08)	0.47 (0.1)	0.68 (0.1)	-
Number of geriatric resources, median (Q1-3)		6 (4–7)	7 (6–8)	6 (5–7)	5 (3–6)	<0.001
Number of geriatric syndromes, median (Q1-3)		6 (4–8)	4 (3–6)	6 (4–7)	8 (7–9)	<0.001
Number of medications, mean (SD)		10.1 (3.6)	8.8 (3.8)	10.5 (3.4)	10.6 (3.3)	<0.001
Living condition, *n* (%)	with relatives	250 (67)	81 (94)	129 (61)	40 (51)	<0.001
	institutionalised/private attendant	34 (9)	1 (1)	15 (7)	18 (23)	
	alone	91 (24)	4 (5)	66 (31)	21 (27)	
Weight loss in the last 3 months, *n* (%)		218 (59)	42 (49)	123 (59)	53 (67)	0.116
BMI, mean (SD)		26.7 (5.6)	26.9 (4.9)	27.2 (5.8)	25.0 (5.5)	0.285
Use of home services, *n* (%)		106 (28)	1 (1)	59 (28)	46 (58)	<0.001
Grade of care (*n* = 495), *n* (%)	none	207 (55)	73 (85)	116 (56)	18 (23)	<0.001
	1–2	103 (28)	8 (9)	68 (33)	27 (34)	
	3	48 (13)	5 (6)	22 (11)	21 (27)	
	4–5	16 (4)	0	3 (1)	13 (17)	
Medical findings						
Renal Replacement Therapy (RRT), *n* (%)		138 (37)	24 (28)	75 (36)	39 (49)	0.181
Kidney-transplant recipients, *n* (%)		45 (12)	15 (18)	23 (11)	7 (9)	0.864
Haemoglobin admission (g/dL), mean (SD)		10.0 (2.2)	10.3 (2.2)	9.9 (2.1)	10.0 (2.2)	0.227
Haemoglobin discharge (g/dL), mean (SD)		10.2 (1.9)	10.7 (1.5)	10.0 (2.1)	9.7 (1.7)	0.316
Total protein admission (g/L), mean (SD)		63.3 (9.6)	65.1 (7.0)	63.3 (9.3)	60.9 (12.4)	0.007
Total protein discharge (g/L), mean (SD)		63.3 (11.0)	66.8 (10.4)	62.5 (11.2)	59.9 (9.9)	0.235
Albumin admission (g/L), mean (SD)		30.9 (6.9)	33.6 (5.7)	31.1 (6.6)	26.9 (7.6)	<0.001
Albumin discharge (g/L), mean (SD)		30.9 (6.8)	34.1 (5.3)	30.7 (6.5)	26.3 (7.8)	<0.001
C-reactive protein admission (mg/dL), median (Q1-3)		32 (12–83)	22 (6–57)	33 (10–75)	50 (22–106)	0.016
C-reactive protein discharge (mg/dL), median (Q1-3)		21 (8–49)	8 (5–39)	22 (9–46)	35 (12–83)	0.020
Proteinuria (g/g Creatinine), median (Q1-3) [*n* = 205]		0.5 (0.2–1.4)	0.6 (0.1–2.4)	0.4 (0.2–0.9)	0.9 (0.4–1.9)	0.521
Albuminuria (g/g Creatinine), median (Q1-3) [*n* = 186]		0.1 (0.0–0.4)	0.1 (0.0–0.4)	0.1 (0.0–0.4)	0.2 (0.1–0.7)	0.667
Alpha-1-Microglobuline Urine (g/g Creatinine), median (Q1-3) [*n* = 189]		0.1 (0.0–0.2)	0.1 (0.0–0.2)	0.1 (0.0–0.2)	0.2 (0.1–0.4)	0.122
Follow-Up 3 months (*n* = 346)						
Alive, *n* (%)		263 (76)	79 (93)	154 (80)	30 (44)	<0.001
Rehospitalisation, (*n* = 271), *n* (%)		160 (59)	45 (56)	90 (57)	25 (78)	0.140
Follow-Up 6 months (*n* = 341)						
Alive, *n* (%)		234 (69)	76 (91)	137 (71)	21 (32)	<0.001
Rehospitalisation, (*n* = 269), *n* (%)		192 (71)	57 (70)	105 (68)	30 (91)	0.027
Follow-Up 12 months (*n* = 306)						
Alive, *n* (%)		174 (57)	58 (75)	100 (61)	16 (25)	0.001
Rehospitalisation, (*n* = 247), *n* (%)		210 (85)	60 (80)	118 (85)	32 (91)	0.109

Notes: LHS = Length of hospital stay; MPI = Multidimensional Prognostic Index; CIRS = Cumulative Illness Rating Scale; ADL = Activities of Daily living; IADL = Instrumental Activities of Daily Living; MNA-SF = Mini Nutritional Assessment-Short form; SPMSQ = Short Portable Mental Status Questionnaire; ESS = Exton Smith Scale ° after linear/logistic regression analysis, results were adjusted for age, gender and KDIGO G-stadium.

**Table 2:** Clinical characteristics of CKD patients according to KDIGO stages.

		Total *n* = 375	G2 (GFR 60–89 mL/min/1.73 m^2^) *n* = 19	G3a (GFR 45–59 mL/min/1.73 m^2^) *n* = 80	G3b (GFR 30–44 mL/min/1.73 m^2^) *n* = 75	G4 (GFR 15–29 mL/min/1.73 m^2^) *n* = 52	G5 (GFR < 15 mL/min/1.73 m^2^) *n* = 149	*p*-Value °
KDIGO stage according to A (*n* = 178), *n* (%)	A1	41 (23.0)	3 (37.5)	21 (42.9)	13 (25.0)	0	4 (8.7)	<0.001
	A2	65 (36.5)	3 (37.5)	17 (34.7)	26 (50.0)	10 (43.5)	9 (19.6)	
	A3	72 (40.4)	2 (25.0)	11 (22.4)	13 (25.0)	13 (56.5)	33 (71.7)	
Demographic								
Age (years), median (IQR)		78.0 (9)	73 (10)	78 (9)	77 (9)	76 (9)	78 (8)	0.121
Female, *n* (%)		133 (35.5)	4 (21.1)	27 (33.8)	32 (42.7)	16 (30.8)	54 (36.2)	0.565
Living conditions, *n* (%)	With relatives	250 (66.7)	15 (78.9)	54 (67.5)	46 (61.3)	34 (65.4)	101 (67.8)	0.117
	Institutionalised/private attendant	34 (9.1)	0	5 (6.3)	6 (8.0)	2 (3.8)	21 (14.1)	
	Alone	91 (24.3)	4 (21.1)	21 (26.3)	23 (30.7)	16 (30.8)	27 (18.1)	
LHS, mean (SD)		14.9 (16.1)	17.1 (23.4)	10.0 (9.6)	11.7 (8.4)	12.0 (7.2)	20.0 (21.1)	<0.001
Body Mass Index (BMI), median (IQR)		25.7 (7)	27.7 (5)	26.4 (8)	26.6 (7)	25.4 (9)	24.8 (7)	0.012
Care								
Grade of Care (*n* = 374), *n* (%)	none	207 (55.3)	13 (68.4)	56 (70.0)	45 (60.0)	34 (65.4)	59 (39.9)	0.091
	1–2	103 (27.5)	5 (26.4)	16 (20.1)	19 (25.4)	15 (28.9)	48 (32.4)	
	3	48 (12.8)	1 (5.3)	6 (7.5)	9 (12.0)	2 (3.8)	30 (20.3)	
	4–5	16 (4.3)	0	2 (2.5)	2 (2.7)	1 (1.9)	11 (7.5)	
Home care, *n* (%)		106 (28.3)	3 (15.8)	12 (15.0)	19 (25.3)	14 (26.9)	58 (38.9)	0.045
Medical findings								
Main Reason for CKD (*n* = 305), *n* (%)	Hypertensive Nephropathy	72 (23.6)	3 (23.1)	17 (29.3)	16 (25.8)	7 (15.9)	29 (22.7)	0.632
	Diabetic Nephropathy	67 (22.0)	2 (15.4)	10 (17.2)	15 (24.2)	12 (27.3)	28 (21.9)	
	Glomerulonephritis	46 (15.1)	4 (30.8)	9 (15.5)	7 (11.3)	6 (13.6)	20 (15.6)	
	Ischemic Disease	37 (12.1)	0	6 (10.3)	12 (19.4)	3 (6.8)	16 (12.5)	
	Other	83 (39.3)	4 (30.8)	16 (27.6)	12 (19.3)	24 (27.3)	35 (27.2)	
Haemoglobin admission (g/dL), median (IQR)		9.8 (2.9)	10.7 (1.4)	10.4 (2.9)	9.6 (2.9)	9.3 (3.2)	9.7 (2.9)	0.045
Haemoglobin discharge (g/dL), median (IQR)		9.8 (2.3)	8.9 (4.2)	10.2 (2.0)	9.8 (1.9)	9.6 (3.5)	10.0 (3.2)	0.781
Total protein admission (g/L), median (IQR)		66.0 (12)	60.5 (7)	68.0 (13	61.5 (11)	62.5 (24)	61.5 (14)	0.728
Total protein discharge (g/L), median (IQR)		64.0 (13)	65.0 (10)	68.0 (8)	63.5 (12)	61.0 (19)	60.0 (12)	0.254
Albumin admission (g/L), median (IQR)		31 (9.75)	31.5 (9)	33.0 (9)	32.0 (8)	32.0 (11)	28.0 (9)	0.049
Albumin discharge (g/L), median (IQR)		32.0 (9)	34.5 (8)	33.5 (10)	31.0 (6)	31.0 (10)	29.0 (8)	0.345
Parathyreoidal hormone admission, median (IQR)		105.5 (153)	81 (101)	110.5 (81)	125 (123)	94 (184)	109 (182)	0.780
Geriatric profile								
MPI, median (IQR)		0.44 (0.30)	0.38 (0.31)	0.44 (0.19)	0.44 (0.19)	0.47 (0.31)	0.50 (0.31)	0.003
MPI group, *n* (%)	MPI-1	86 (22.9)	8 (42.1)	23 (28.7)	14 (18.7)	17 (32.7)	24 (16.1)	0.018
	MPI-2	210 (56.0)	9 (47.4)	46 (57.5)	50 (66.7)	23 (44.2)	82 (55.0)	
	MPI-3	79 (21.1)	2 (10.5)	11 (13.8)	11 (14.7)	12 (23.1)	43 (28.9)	
Geriatric resources (GR), mean (SD)		5.8 (2.1)	6.1 (1.9)	6.6 (1.9)	5.7 (2.0)	5.8 (2.2)	5.5 (2.1)	0.006
Geriatric resources (extract)								
Physical, *n* (%)		102 (27.2)	10 (52.6)	30 (37.5)	17 (22.7)	11 (21.2)	34 (22.8)	0.030
Good living conditions, *n* (%)		259 (69.3)	10 (52.6)	63 (79.7)	49 (65.3)	29 (55.8)	108 (72.5)	0.007
Social resources, *n* (%)		323 (86.4)	13 (68.4)	76 (96.2)	60 (80.0)	43 (82.7)	131 (87.9)	0.003
Geriatric Syndromes (GS), mean (SD)		5.8 (2.4)	5.4 (2.6)	5.2 (2.4)	6.0 (2.1)	5.6 (2.5)	6.3 (2.3)	0.499
Geriatric syndromes (extract)								
Inanition, *n* (%)		183 (48.8)	10 (52.6)	34 (42.5)	30 (40.0)	19 (36.5)	90 (60.4)	0.030
Polypharmacy, *n* (%)		341 (90.9)	15 (78.9)	65 (81.3)	66 (88.0)	51 (98.1)	144 (96.6)	<0.001
Follow-Up Discharge								
Alive, *n* (%)		352 (93.9)	19 (100)	75 (93.8)	73 (97.3)	48 (92.3)	137 (91.9)	0.450
MPI, median (IQR)		0.44 (0.19)	0.38 (0.25)	0.38 (0.19)	0.44 (0.19)	0.38 (0.30)	0.50 (0.25)	<0.001
MPI (*n* = 349), *n* (%)	MPI-1	82 (23.5)	9 (47.4)	25 (33.3)	14 (19.4)	15 (31.3)	19 (14.1)	<0.001
	MPI-2	222 (63.6)	9 (47.4)	43 (57.3)	50 (69.4)	25 (52.)	95 (70.4)	
	MPI-3	45 (12.9)	1 (5.3)	7 (9.3)	8 (11.1)	8 (16.7)	21 (15.6)	
Follow-Up 3 months (*n* = 346)								
Alive, *n* (%)		263 (76.0)	17 (94.4)	64 (83.1)	54 (78.3)	36 (73.5)	92 (69.2)	0.318
Grade of care, (*n* = 246), *n* (%)		122 (49.6)	6 (37.5)	25 (44.6)	26 (50.0)	13 (36.1)	52 (60.5)	0.574
Rehospitalisation, (*n* = 271), *n* (%)		160 (59.0)	5 (29.4)	33 (52.4)	32 (59.3)	26 (66.7)	64 (65.3)	0.081
Follow-Up 6 months (*n* = 341)								
Alive, *n* (%)		234 (68.6)	17 (94.4)	58 (77.3)	46 (67.6)	33 (67.3)	80 (61.6)	0.124
Grade of care, (*n* = 221), *n* (%)		120 (54.3)	9 (52.9)	19 (35.8)	27 (60.0)	9 (30.0)	56 (73.7)	<0.001
Rehospitalisation, (*n* = 269), *n* (%)		192 (71.4)	8 (47.1)	38 (61.3)	41 (75.9)	29 (76.3)	76 (77.6)	0.038
Follow-Up 12 months (*n* = 306)								
Alive, *n* (%)		174 (56.9)	13 (81.3)	43 (63.2)	34 (55.7)	26 (57.8)	58 (50.0)	<0.001
Grade of care, (*n* = 169), *n* (%)		95 (56.2)	9 (69.2)	14 (35.0)	21 (61.8)	11 (42.3)	40 (71.4)	0.003
Rehospitalisation, (*n* = 247), *n* (%)		210 (85.0)	9 (64.3)	40 (70.2)	45 (90.0)	31 (86.1)	85 (94.4)	<0.001

Notes: Patients were subdivided into KDIGO-G group after the known prehospital diagnosis of CKD KDIGO, all patients being under RRT were classified as stage G5. If information according to KDIGO group was missing, the best achieved GFR admission or discharge was used for classification. If there was one information missing of GFR admission or discharge, patients were excluded from analysis. LHS = Length of hospital stay; MPI = Multidimensional Prognostic Index; CIRS = Cumulative Illness Rating Scale; ADL= Activities of Daily living; IADL = Instrumental Activities of Daily Living; MNA-SF = Mini Nutritional Assessment-Short form; SPMSQ = Short Portable Mental Status Questionnaire; ESS = Exton Smith Scale; ° after linear/logistic regression analysis, results were adjusted for age, gender and MPI.

**Table 3:** Clinical characteristics of RRT patients according to different types of RRT.

		Total *n* = 138	HD *n* = 127	PD *n* = 11	*p*-Value °
Age (years), median (IQR)		78 (8)	78.0 (9)	78.0 (5)	0.289
Female, *n* (%)		49 (37.5)	47 (37.0)	2 (18.2)	0.185
Hospitalisation last 12 months, *n* (%)		99 (71.7)	90 (70.9)	9 (81.8)	0.311
LHS (days), median (IQR)		15 (19)	15 (18)	11.0 (23)	0.818
BMI (kg/m^2^), median (IQR))		24.7 (6.3)	24.8 (6.3)	23.9 (5.3)	0.716
Haemoglobin at admission (g/L)		9.7 (3.0)	9.7 (2.7)	9.4 (2.7)	0.402
Albumin at admission (g/L)		38.5 (9)	29.0 (12)	27 (5.3)	0.367
Living conditions, *n* (%)	With relatives	95 (68.8)	86 (67.7)	9 (81.8)	0.664
	Institutionalised/private attendant	19 (13.8)	18 (14.2)	1 (9.1)	
	Alone	24 (17.4)	23 (18.1)	1 (9.1)	
Grade of Care, *n* (%)	none	58 (42.0)	50 (39.4)	8 (72.7)	0.478
	1–2	43 (31.1)	41 (32.2)	2 (18.2)	
	3	27 (19.6)	26 (20.5)	1 (9.1)	
	4–5	10 (7.2)	10 (7.9)	0	
Home care, *n* (%)		51 (37.0)	49 (38.6)	2 (18.2)	0.218
MPI, median (IQR)		0.50 (0.31)	0.53 (0.31)	0.49 (0.17)	0.414
MPI group at admission, *n* (%)	MPI-1	24 (17.4)	22 (17.3)	2 (18.2)	0.316
	MPI-2	75 (54.3)	67 (52.8)	8 (72.7)	
	MPI-3	39 (28.3)	38 (29.9)	1 (9.1)	
CIRS, mean (SD)		5.6 (1.6)	5.6 (1.5)	4.6 (1.4)	0.026
ESS, mean (SD)		14.2 (3.4)	14.1 (3.5)	15.3 (2.4)	0.688
MNA-SF, mean (SD))		8.2 (3.3)	8.2 (3.2)	8.3 (4.1)	0.503
ADL, mean (SD)		3.6 (2.0)	3.6 (2.1)	4.2 (1.5)	0.892
IADL, mean (SD)		4.1 (2.5)	4.0 (2.4)	4.9 (2.7)	0.498
SPMSQ, mean (SD)		1.9 (1.9)	1.9 (1.9)	1.1 (1.6)	0.199
Geriatric resources (GR), mean (SD)		5.5 (2.0)	5.5 (4.0)	5.8 (1.8)	0.848
Emotional, *n* (%)		88 (63.8)	78 (61.4)	10 (90.9)	0.045
Geriatric Syndromes (GS), mean (SD)		6.4 (2.3)	6.4 (2.3)	5.6 (1.8)	0.472
Sensorial Impairment, *n* (%)		86 (62.3)	83 (65.4)	3 (27.3)	0.019
Irritability/Depression, *n* (%)		21 (15.2)	21 (16.5)	0	0.073
Insomnia, *n* (%)		76 (55.1)	69 (54.3)	7 (63.6)	0.487
Alive, *n* (%)		129 (93.5)	119 (93.7)	10 (90.9)	0.529
MPI, mean (SD)		0.54 (0.17)	0.54 (0.17)	0.41 (0.15)	0.021
Alive, *n* (%)		85 (69.7)	77 (68.8)	8 (80.0)	0.885
Grade of Care, *n* (%)		47 (58.8)	44 (61.1)	3 (37.5)	0.260
Rehospitalisation, *n* (%)		60(65.9)	55 (67.1)	5 (55.6)	0.596
Alive, *n* (%)		74 (61.7)	67 (60.9)	7 (70.0)	0.987
Grade of Care, *n* (%)		51 (71.8)	47 (74.6)	4 (50.0)	0.194
Rehospitalisation, *n* (%)		71 (78.0)	65 (79.3)	6 (66.7)	0.636
Alive, *n* (%)		53 (50.0)	49 (50.0)	4 (50.0)	0.691
Grade of Care, *n* (%)		36 (69.2)	35 (72.9)	1 (25.0)	0.083
Rehospitalisation, *n* (%)		80 (95.2)	73 (94.8)	7 (100)	0.332

Notes: PD = Peritoneal Dialysis, HD = Haemodialysis; LHS = Length of hospital stay; MPI = Multidimensional Prognostic Index; CIRS = Cumulative Illness Rating Scale; ADL= Activities of Daily living; IADL = Instrumental Activities of Daily Living; MNA-SF = Mini Nutritional Assessment-Short form; SPMSQ = Short Portable Mental Status Questionnaire; ESS = Exton Smith Scale; ° between the different renal replacement therapy (RRT), after testing for normal distribution (Kolmogorov–Smirnov), a Kruskal–Wallis test was used for ordinary or nominal parameters; Chi-squared or Fisher’s Exact tests were used for frequencies. After linear/logistic regression analysis, results were adjusted for MPI except for MPI and MPI group. No results are adjusted if not otherwise specified, significant at 5%.

**Table 4:** The 12-month outcomes for kidney transplant recipients (KTR) compared to patients undergoing renal replacement therapy (RRT).

		Total *n* = 175	Renal Replacement Therapy (RRT) *n* = 131	Kidney Transplant Recipients *n* = 44	*p*-Value °
Age (years), median (IQR).		77 (9)	78.0 (8)	73.0 (7)	<0.001
Female, *n* (%)		61 (35)	48 (37)	13 (30)	0.307
Period of education, mean (SD)		12.4 (4.0)	11.9 (3.5)	13.7 (5.1)	0.004
Hospitalisation last 12 months, *n* (%)		130 (74)	95 (73)	35 (80)	0.900
Falls last 12 months, *n* (%)		76 (43)	66 (50)	10 (22)	0.014
LHS (days), mean (SD)		18.9 (20.8)	19.9 (21.8)	14.0 (17.1)	0.077
BMI (kg/m^2^), median (IQR)		25.7 (6.95)	24.8 (6.3)	25.4 (6.3)	<0.001
Living conditions, *n* (%)	With relatives	121 (69)	90 (69)	31 (71)	0.244
	Institutionalised/private attendant	21 (12)	19 (15)	2 (5)	
	Alone	33 (19)	22 (17)	11 (25)	
Grade of Care, *n* (%)	none	85 (48.6)	54 (41)	31 (71)	0.031
	1–2	50 (29)	41 (31)	9 (20)	
	3	30 (17)	26 (20)	4 (9)	
	4–5	10 (6)	10 (8)	0	
Home care, *n* (%)		61 (35)	51 (39)	10 (23)	0.751
MPI, mean (SD)		0.51 (0.18)	0.52 (0.17)	0.43 (0.16)	0.028
MPI group, *n* (%)	MPI-1	39 (22)	23 (18)	16 (36)	0.038
	MPI-2	91 (52)	70 (53)	21 (48)	
	MPI-3	45 (26)	38 (29)	7 (16)	
Geriatric resources, median (IQR)		6.0 (3)	6.0 (3)	6.0 (3)	0.664
Geriatric Syndromes, median (IQR)		6.0 (4)	6.0 (4)	5.0 (4)	0.106
Immobility, *n* (%)		86 (49)	70 (53)	16 (36)	0.101
Sensorial Impairment, *n* (%)		103 (59)	83 (63)	20 (46)	0.169
Inanition, *n* (%)		97 (55)	80 (61)	17 (39)	0.565
Instability, *n* (%)		116 (66)	95 (73)	21 (48)	0.439
Insomnia, *n* (%)		90 (51)	73 (56)	17 (39)	0.044
More GR than GS, *n* (%)		120 (69)	85 (65)	35 (80)	0.349
Follow-Up Discharge (*n* = 175)					
Alive, *n* (%)		163 (93)	122 (93)	42 (93)	0.518
MPI, mean (SD)		0.51 (0.18)	0.50 (0.15)	0.42 (0.15)	0.110
Falls, *n* (%)		11 (7)	8 (7)	3 (7)	0.787
Follow-Up 3 months (*n* = 157)					
Alive, *n* (%)		113 (72)	79 (69)	34 (81)	0.523
Grade of Care, *n* (%)		56 (53)	44 (60)	12 (38)	0.333
Rehospitalisation, *n* (%)		77 (65)	56 (66)	21 (64)	0.725
Falls, *n* (%)		31 (28)	25 (32)	6 (18)	0.253
Follow-Up 6 months (*n* = 155)					
Alive, *n* (%)		102 (66)	69 (61)	33 (79)	0.368
Grade of Care, *n* (%)		57 (60)	47 (72)	10 (33)	0.015
Rehospitalisation, *n* (%)		94 (80)	67 (79)	27 (82)	0.865
Falls, *n* (%)		37 (37)	29 (42)	8 (26)	0.207
Follow-Up 12 months (*n* = 141)					
Alive, *n* (%)		78 (55)	49 (49)	29 (71)	0.395
Grade of Care, *n* (%)		43 (59)	34 (71)	9 (36)	0.054
Rehospitalisation, *n* (%)		103 (93)	76 (96)	27 (84)	0.010
Falls, *n* (%)		37 (42)	29 (48)	8 (29)	0.999

Notes: PD = Peritoneal Dialysis, HD = Haemodialysis; LHS = Length of hospital stay; MPI = Multidimensional Prognostic Index; CIRS = Cumulative Illness Rating Scale; ADL = Activities of Daily living; IADL = Instrumental Activities of Daily Living; MNA-SF = Mini Nutritional Assessment-Short form; SPMSQ = Short Portable Mental Status Questionnaire; ESS= Exton Smith Scale; ° between the different RRT (HD with Shunt, HD with catheter, PD), after testing for normal distribution (Kolmogorov–Smirnov), a Kruskal–Wallis test was used for ordinary or nominal parameters; Chi-squared or Fisher’s Exact tests were used for frequencies. After linear/logistic regression analysis, results were adjusted for age and MPI. No results are adjusted if not otherwise specified, significant at 5%.

**Table 5:** The 12-month outcomes for patients with CKD G4-5 without renal replacement therapy (RRT) vs. patients with CKD G5 and haemodialysis (HD).

		Total *n* = 190	CKD G4-5 Non-RRT *n* = 52	Haemodialysis *n* = 138	*p*-Value °
Age (years), median (IQR)		78.0 (9)	76.0 (8)	78.0 (9)	0.027
Female, *n* (%)		68 (36)	19 (37)	49 (36)	0.732
LHS (days), mean (SD)		18.1 (19.1)	12.0 (7.0)	20.4 (21.6)	0.003
BMI (kg/m^2^), median (IQR)		25.1 (7.4)	25.9 (9.7)	24.9 (6.9)	0.046
Albumin at admission, median (IQR)			32 (12)	28.5 (11)	0.011
Living conditions, *n* (%)	With relatives	126 (66)	34 (65)	92 (67)	0.261
	Institutionalised/private attendant	22 (12)	3 (6)	19 (14)	
	Alone	42 (22)	15 (29)	27 (20)	
Grade of Care, *n* (%)	none	85 (45)	32 (62)	53 (39)	<0.001
	1–2	61 (32)	15 (29)	46 (34)	
	3	31 (16)	4 (8)	27 (20)	
	4–5	12 (6)	1 (2)	11 (8)	
Home care, *n* (%)		70 (37)	15 (29)	55 (40)	<0.001
MPI, mean (SD)		0.52 (0.17)	0.47 (0.18)	0.53 (0.16)	0.052
MPI group, *n* (%)	MPI-1	39 (21)	17 (33)	22 (16)	0.018
	MPI-2	97 (51)	23 (44)	74 (54)	
	MPI-3	54 (28)	12 (23)	42 (30)	
Geriatric resources, mean (SD)		5.5 (2.1)	5.7 (2.3)	5.5 (2.1)	0.355
Geriatric Syndromes, median (IQR)		6.0 (4)	5.0 (3)	6.0 (3)	0.044
Follow-Up Discharge (*n* = 190)					
Alive, *n* (%)		175 (92)	47 (90)	128 (93)	0.460
MPI, mean (SD)		0.50 (0.16)	0.44 (0.17)	0.52 (0.15)	0.005
Follow-Up 3 months (*n* = 172)					
Alive, *n* (%)		120 (70)	36 (74)	84 (68)	0.186
Grade of Care, (*n* = 114) *n* (%)		62 (54)	13 (37)	49 (62)	0.010
Rehospitalisation, *n* (%)		85 (66)	25 (66)	60 (67)	0.839
Follow-Up 6 months (*n* = 170)					
Alive, *n* (%)		106 (62)	34 (69)	72 (60)	0.133
Grade of Care, (*n* = 98) *n* (%)		61 (62)	10 (32)	51 (76)	0.076
Rehospitalisation (*n* = 172), *n* (%)		99 (78)	28 (76)	71 (79)	0.862
Follow-Up 12 months (*n* = 153)					
Alive, *n* (%)		80 (52)	28 (62)	52 (48)	0.039
Grade of Care, (*n* = 78) *n* (%)		50 (64)	13 (46)	37 (74)	0.003
Rehospitalisation, (*n* = 119) *n* (%)		109 (92)	30 (86)	79 (94)	0.334

Notes: PD = Peritoneal Dialysis, HD = Haemodialysis; LHS = Length of hospital stay; MPI = Multidimensional Prognostic Index; CIRS = Cumulative Illness Rating Scale; ADL = Activities of Daily living; IADL = Instrumental Activities of Daily Living; MNA-SF = Mini Nutritional Assessment-Short form; SPMSQ = Short Portable Mental Status Questionnaire; ESS = Exton Smith Scale; ° between the different RRT (HD with Shunt, HD with catheter, PD), after testing for normal distribution (Kolmogorov–Smirnov), a Kruskal–Wallis test was used for ordinary or nominal parameters; Chi-squared or Fisher’s Exact tests were used for frequencies. *p*-value after linear/logistic regression analysis, results were adjusted for age. No results are adjusted if not otherwise specified, significant at 5%.

## Data Availability

Data are available on request due to privacy/ethical restrictions.
